# The classification of feeding and eating disorders in the ICD-11: results of a field study comparing proposed ICD-11 guidelines with existing ICD-10 guidelines

**DOI:** 10.1186/s12916-019-1327-4

**Published:** 2019-05-14

**Authors:** Angélica M. Claudino, Kathleen M. Pike, Phillipa Hay, Jared W. Keeley, Spencer C. Evans, Tahilia J. Rebello, Rachel Bryant-Waugh, Yunfei Dai, Min Zhao, Chihiro Matsumoto, Cecile Rausch Herscovici, Blanca Mellor-Marsá, Anne-Claire Stona, Cary S. Kogan, Howard F. Andrews, Palmiero Monteleone, David Joseph Pilon, Cornelia Thiels, Pratap Sharan, Samir Al-Adawi, Geoffrey M. Reed

**Affiliations:** 1 Department of Psychiatry, Universidade Federal de São Paulo (UNIFESP), Rua Major Maragliano, 241, São Paulo, SP 04017-030 Brazil; 2 Department of Psychiatry, Columbia University, Vagelos College of Physicians and Surgeons, Unit 9 Room 5808, 1051 Riverside Drive, New York, NY 10032 USA; 3 Translational Health Research Institute (THRI), School of Medicine, Western Sydney University, Locked Bag 1797, Penrith South, 2751 NSW Campbelltown Australia; 4 Department of Psychology, Virginia Commonwealth University, 806 West Franklin St, Box 842018, Richmond, VA 23284 USA; 5 Department of Psychology, Harvard University, 33 Kirkland St, 1040 William James Hall, Cambridge, MA 02138 USA; 6 Global Mental Health Program, Columbia University College of Physicians and Surgeons and New York State Psychiatric Institute, Mailman School of Public Health, 722 West 168th, Floor R2, R-233, New York, NY 10032 USA; 7 Feeding and Eating Disorders Service, Department of Child and Adolescent Mental Health, Great Ormond Street Hospital for Children NHS Foundation Trust, Population, Policy and Practice Programme, UCL Great Ormond Street Institute of Child Health, 30 Guilford St, London, WC1N 1EH UK; 8 Shanghai Mental Health Center, Shanghai Jiao Tong University School of Medicine, 600 Wan Ping Nan Road, Shanghai, 200030 People’s Republic of China; 9 National Study Coordinator for ICD-11 Field Studies, ICD-11 Committee, Japanese Society of Psychiatry and Neurology, Hongo-Yumicho Building, 2-38-4, Hongo, Bunkyo-ku, Tokyo, 113-0033 Japan; 10 International Life Sciences Institute (ILSI, Argentina), J. Salguero 2745, Buenos Aires, 1425 CABA Argentina; 11 Department of Psychiatry, Faculty of Medicine, Universidad Autónoma de Madrid, Instituto de Salud Carlos III, Centro de Investigación en Red de Salud Mental, 2ª Planta Norte, Calle del Prof Martín Lagos, s/n, 28040 Madrid, Spain; 12 Ministry for Solidarity and Health, Avenue Duquesne, 75350 Paris, France; 13 School of Psychology, Faculty of Social Sciences, 136 Jean-Jacques Lussier, Vanier Hall, Ottawa, ON K1N 6N5 Canada; 14 Departments of Biostatistics and Psychiatry and New York State Psychiatric Institute, Columbia University, Vagelos College of Physicians and Surgeons, Unit 47, 1051 Riverside Drive, New York, NY 10032 USA; 15 Department of Psychiatry, University of Campania “L. Vanvitelli”, Naples, Italy; 16 Department of Medicine, Surgery and Dentistry “Scuola Medica Salernitana”, University of Salerno, via Allende, Baronissi 84081 Salerno, Italy; 17 Nova Scotia Health Authority, Dalhousie University, LeMarchant Place, 2nd Floor, Rm 2121, 1246 LeMarchant Street, Halifax, NS B3H 4R2 Canada; 18 Department of Social Studies, University of Applied Sciences Bielefeld, Kissinger Str. 14, D-12157 Berlin, Germany; 19 Department of Psychiatry, All India Institute of Medical Sciences, Ansari Nagar, New Delhi, 110029 India; 20 Department of Behavioral Medicine, College of Medicine and Health Sciences, Sultan Qaboos University, P.O. Box 35, P.C. 123, Al Khoud, Muscat, Sultanate of Oman; 21 Department of Mental Health and Substance Abuse, World Health Organization, Geneva, Switzerland; 22 Department of Psychiatry, Columbia University, Vagelos College of Physicians and Surgeons, Unit 9 Room 5816, 1051 Riverside Drive, New York, NY 10032 USA

**Keywords:** Eating disorders, Feeding disorders, Diagnosis and classification, Clinical utility, Anorexia nervosa, Bulimia nervosa, Binge eating disorder, Avoidant-restrictive food intake disorder, International classification of diseases, ICD-11

## Abstract

**Background:**

The World Health Organization (WHO) International Classification of Diseases and Related Health Problems (ICD) is used globally by 194 WHO member nations. It is used for assigning clinical diagnoses, providing the framework for reporting public health data, and to inform the organization and reimbursement of health services. Guided by overarching principles of increasing clinical utility and global applicability, the 11th revision of the ICD proposes major changes that incorporate empirical advances since the previous revision in 1992. To test recommended changes in the Mental, Behavioral, and Neurodevelopmental Disorders chapter, multiple vignette-based case-controlled field studies have been conducted which examine clinicians’ ability to accurately and consistently use the new guidelines and assess their overall clinical utility. This manuscript reports on the results from the study of the proposed ICD-11 guidelines for feeding and eating disorders (FEDs).

**Method:**

Participants were 2288 mental health professionals registered with WHO’s Global Clinical Practice Network. The study was conducted in Chinese, English, French, Japanese, and Spanish. Clinicians were randomly assigned to apply either the ICD-11 or ICD-10 diagnostic guidelines for FEDs to a pair of case vignettes designed to test specific clinical questions. Clinicians selected the diagnosis they thought was correct for each vignette, evaluated the presence of each essential feature of the selected diagnosis, and the clinical utility of the diagnostic guidelines.

**Results:**

The proposed ICD-11 diagnostic guidelines significantly improved accuracy for all FEDs tested relative to ICD-10 and attained higher clinical utility ratings; similar results were obtained across all five languages. The inclusion of binge eating disorder and avoidant-restrictive food intake disorder reduced the use of residual diagnoses. Areas needing further refinement were identified.

**Conclusions:**

The proposed ICD-11 diagnostic guidelines consistently outperformed ICD-10 in distinguishing cases of eating disorders and showed global applicability and appropriate clinical utility. These results suggest that the proposed ICD-11 guidelines for FEDs will help increase accuracy of public health data, improve clinical diagnosis, and enhance health service organization and provision. This is the first time in the revision of the ICD that data from large-scale, empirical research examining proposed guidelines is completed in time to inform the final diagnostic guidelines.

## Introduction

Improving diagnostic guidelines for feeding and eating disorders (FEDs) in ICD-11 has significant implications for prevention and treatment. These disorders have a lifetime prevalence above 10% [1] and a point prevalence of at least 5% [2] and rates are increasing in many parts of the world [3–5]. Eating disorders (EDs) are associated with elevated rates of morbidity and mortality [6–9]. Anorexia nervosa (AN) has one of the highest mortality rates of all mental disorders [9]. Individuals with eating disorders have an elevated risk of dying by suicide compared to age-matched population estimates [7, 10]. As measured by the combination of years of life lost due to premature mortality and years lived with disability, the global disease burden of eating disorders increased by 65% between 1990 and 2016 [11]. Given the prevalence, severity, burden, and risk of mortality associated with eating disorders, increasing rates of eating disorders in various regions of the world and, given emerging data on feeding disorders, developing more accurate and clinically useful tools for the identification of such conditions to facilitate prevention and promote effective intervention are important global health priorities.

Feeding and eating disorders are conditions that involve abnormal eating or feeding behaviors that are not better accounted for by other health conditions and are not developmentally appropriate or culturally sanctioned. Feeding disorders include a range of conditions characterized by restricted or limited intake (avoidant-restrictive food intake disorder), as well as behavioral disturbances such as eating of non-edible substances (pica) or voluntary regurgitation of foods (rumination-regurgitation disorder). Eating disorders, i.e., anorexia nervosa, bulimia nervosa (BN), and binge eating disorder (BED), are conditions that are characterized by abnormal eating behaviors, as well as to varying degrees by preoccupation with food, body weight, and shape.

It has been more than 25 years since the World Health Organization (WHO) published the last major revision of the International Classification of Diseases and Related Health Problems (ICD) [12]. Since then, empirical research and evidence-informed clinical practice for eating disorders have evolved dramatically. Corresponding research in the field of feeding disorders has lagged behind, resulting in far less by way of evolution of evidence-informed practice for these disorders. This article describes the findings from a field study comparing the accuracy and consistency of clinician-assigned diagnoses when applying the proposed ICD-11 diagnostic guidelines for eating disorders as compared to the existing ICD-10 diagnostic guidelines to standardized case material. The study also compared clinician ratings of the clinical utility of the proposed guidelines for ICD-11 to those for ICD-10.

In developing the ICD-11 chapter on Mental, Behavioral, and Neurodevelopmental Disorders, the WHO Department of Mental Health and Substance Abuse identified clinical utility and global applicability as guiding principles [13]. To this end, a Working Group convened by WHO reviewed the extant research base on feeding and eating disorders and proposed changes to the ICD-10 guidelines with the following aims: (a) to improve communication among users (e.g., practitioners, patients, families, administrators), (b) to foster conceptualization and understanding of feeding and eating disorders, (c) to accurately and easily describe actual clinical presentations, (d) to assist with clinical management, and (e) to enhance clinical outcomes at the individual and population levels [14].

The Working Group identified three overarching limitations inherent to the ICD-10 eating disorders guidelines [15, 16]: (1) the ICD-10’s separation of feeding and eating disorders into two separate groups is not consistent with empirical data and current clinical practice, (2) the ICD-10 guidelines result in a lack of consistency in assigned diagnoses for eating disorders, with a large proportion classified using available “atypical” categories or “other specified” or “unspecified” residual categories, and (3) the ICD-10 guidelines fail to explicitly recognize the full range of cultural differences in clinical manifestations of feeding and eating disorders.

To address the first shortcoming of the ICD-10, and consistent with the Diagnostic and Statistical Manual of Mental Disorders 5th Edition (DSM-5) [17], feeding and eating disorders represent a single grouping in the ICD-11 (Table 1 summarizes the essential features of proposed categories). Further, to improve the clinical utility of the diagnostic system and to reduce the use of “atypical,” “other specified,” or “unspecified” diagnostic categories in ICD-10, which have limited clinical utility or informational value, the Working Group recommended (1) broadening the guidelines for AN and BN to include atypical and developmental variations of presentation, (2) adding BED, and (3) adding avoidant-restrictive food intake disorder (ARFID) to the diagnostic nomenclature [16]. To a great extent, the addition of ARFID represents a revised and expanded understanding of F98.2 Feeding disorder of infancy and childhood [18].

**Table 1:** Proposed ICD-11 diagnostic guidelines (essential features only) for feeding and eating disorders after revisions based on the study result

Anorexia nervosa	
*Essential (required) features:* • Significantly low body weight for the individual’s height, age, developmental stage and weight history that is not due to the unavailability of food and is not better accounted for by another medical condition. A commonly used guideline is body mass index (BMI) less than 18.5 kg/m^2^ in adults and BMI-for-age under 5th percentile in children and adolescents. Rapid weight loss (e.g., more than 20% of total body weight within 6 months) may replace the low body weight guideline as long as other diagnostic requirements are met. Children and adolescents may exhibit failure to gain weight as expected based on the individual developmental trajectory rather than weight loss. • A persistent pattern of restrictive eating or other behaviors that are aimed at establishing or maintaining abnormally low body weight, typically associated with extreme fear of weight gain. Behaviors may be aimed at reducing energy intake, by fasting, choosing low calorie food, excessively slow eating of small amounts of food, and hiding or spitting out food, as well as purging behaviors, such as self-induced vomiting and use of laxatives, diuretics, enemas, or omission of insulin doses in individuals with diabetes. Behaviors may also be aimed at increasing energy expenditure through excessive exercise, motor hyperactivity, deliberate exposure to cold, and use of medication that increases energy expenditure (e.g., stimulants, weight loss medication, herbal products for reducing weight, thyroid hormones). • Low body weight is overvalued and central to the person’s self-evaluation, or the person’s body weight or shape is inaccurately perceived to be normal or even excessive. Preoccupation with weight and shape, when not explicitly stated, may be manifested by behaviors such as repeatedly checking body weight using scales, checking one’s body shape using tape measures or reflection in mirrors, constant monitoring of the calorie content of food and searching for information on how to lose weight or by extreme avoidant behaviors, such as refusal to have mirrors at home, avoidance of tight-fitting clothes, or refusal to know one’s weight or purchase clothing with specified sizing.	
Bulimia nervosa	
*Essential (required) features:* • Frequent, recurrent episodes of binge eating (e.g., once a week or more over a period of at least 1 month). Binge eating is defined as a distinct period of time during which the individual experiences a loss of control over his or her eating behavior. A binge eating episode is present when an individual eats notably more and/or differently than usual and feels unable to stop eating or limit the type or amount of food eaten. Other characteristics of binge eating episodes may include eating alone because of embarrassment, eating foods that are not part of the individual’s regular diet, eating large amounts of food in spite of not feeling hungry, and eating faster than usual. • Repeated inappropriate compensatory behaviors to prevent weight gain (e.g., once a week or more over a period of at least 1 month). The most common compensatory behavior is self-induced vomiting, which typically occurs within an hour of binge eating. Other inappropriate compensatory behaviors include fasting or using diuretics to induce weight loss, using laxatives or enemas to reduce the absorption of food, omission of insulin doses in individuals with diabetes, and strenuous exercise to greatly increase energy expenditure. • Excessive preoccupation with body weight and shape. When not explicitly stated, preoccupation with weight and shape may be manifested by behaviors such as repeatedly checking body weight using scales, checking one’s body shape using tape measures or reflection in mirrors, constant monitoring of the calorie content of food and searching for information on how to lose weight or by extreme avoidant behaviors, such as refusal to have mirrors at home, avoidance of tight-fitting clothes, or refusal to know one’s weight or purchase clothing with specified sizing. • There is marked distress about the pattern of binge eating and inappropriate compensatory behavior or significant impairment in personal, family, social, educational, occupational or other important areas of functioning. • The symptoms do not meet the definitional requirements for Anorexia Nervosa.	
Binge eating disorder	
*Essential (required) features:* • Frequent, recurrent episodes of binge eating (e.g., once a week or more over a period of 3 months). Binge eating is defined as a distinct period of time during which the individual experiences a loss of control over his or her eating behavior. A binge eating episode is present when an individual eats notably more or differently than usual and feels unable to stop eating or limit the type or amount of food eaten. Other characteristics of binge eating episodes may include eating alone because of embarrassment, or eating foods that are not part of the individual’s regular diet. • The binge eating episodes are not regularly accompanied by inappropriate compensatory behaviors aimed at preventing weight gain. • The symptoms and behaviors are not better explained by another medical condition (e.g., Prader-Willi Syndrome) or another mental disorder (e.g., a depressive disorder) and are not due to the effect of a substance or medication on the central nervous system, including withdrawal effects. • There is marked distress about the pattern of binge eating or significant impairment in personal, family, social, educational, occupational or other important areas of functioning.	
Avoidant-restrictive food intake disorder	
*Essential (required) features:* • Avoidance or restriction of food intake that results in either or both of the following: o The intake of an insufficient quantity or variety of food to meet adequate energy or nutritional requirements that has resulted in significant weight loss, clinically significant nutritional deficiencies, dependence on oral nutritional supplements or tube feeding, or has otherwise negatively affected the physical health of the individual. o Significant impairment in personal, family, social, educational, occupational or other important areas of functioning (e.g., due to avoidance or distress related to participating in social experiences involving eating). • The pattern of eating behavior is not motivated by preoccupation with body weight or shape or by significant body image distortion. • Restricted food intake and consequent weight loss (or failure to gain weight) or other impact on physical health is not due to unavailability of food, not a manifestation of another medical condition (e.g., food allergies, hyperthyroidism), and not due to the effect of a substance or medication (e.g., amphetamine), including withdrawal, and not due to another mental disorder.	
Pica	
*Essential (required) features:* • Regular consumption of non-nutritive substances, such as non-food objects and materials (e.g., clay, soil, chalk, plaster, plastic, metal and paper), or raw food ingredients (e.g., large quantities of salt or corn flour). • The ingestion of non-nutritive substances is persistent or severe enough to require clinical attention. That is, the behavior causes damage to health, impairment in functioning, or significant risk due to the frequency, amount or nature of the substances or objects ingested. • Based on age and level of intellectual functioning, the individual would be expected to distinguish between edible and non-edible substances. In typical development, this occurs at approximately 2 years of age. • The symptoms or behaviors are not a manifestation of another medical condition (e.g., nutritional deficiency).	
Rumination-regurgitation disorder	
*Essential (required) features:* • The intentional and repeated bringing up of previously swallowed food back to the mouth (i.e., regurgitation), which may be re-chewed and re-swallowed (i.e., rumination), or may be deliberately spat out (but not as in vomiting). • The regurgitation behavior is frequent (at least several times per week) and sustained over a period of at least several weeks. • The diagnosis should only be assigned to individuals who have reached a developmental age of at least 2 years. • The regurgitation behavior is not a manifestation of another medical condition that directly causes regurgitation (e.g., esophageal strictures or neuromuscular disorders affecting esophageal functioning) or causes nausea or vomiting (e.g., pyloric stenosis).	

Eight specific research questions that are the focus of the present study emerged as a result of the recommended changes in the ICD for feeding and eating disorders. These questions represent fundamental conceptual changes made to the classification on the basis of a rigorous review of the empirical literature, including cross-culturally. Because the diagnostic guidelines for pica and rumination-regurgitation disorder had not changed substantially, these diagnoses were not included in the present study. Our overarching hypothesis was that revisions made to render the ICD-11 diagnostic guidelines more consistent with current research and to increase its clinical utility and global applicability would improve clinicians’ diagnostic accuracy and consistency when using the proposed ICD-11 guidelines for eating disorders, and that clinicians would rate the ICD-11 diagnostic guidelines as more clinically useful, as compared to those using the existing ICD-10 guidelines.

## Methods

### Description of study design

This was an experimental, vignette-based case-controlled study implemented via the internet with participation from a large, global, multilingual, and multidisciplinary sample of mental health professionals. The current study is part of a larger research program that employs a standard research design across the range of mental and behavioral disorders to assess the impact and clinical utility of proposed changes in the ICD guidelines. Additional information about the rationale and experimental design for these studies has been published elsewhere [19, 20].

### Eight core questions

The eight core research questions investigated in this study were as follows:

1. Does the proposed addition of ARFID in the ICD-11 result in individuals with ARFID being more accurately distinguished from AN, and does the proposed addition of ARFID to ICD-11 reduce the number of individuals diagnosed with residual eating disorder categories (atypical, other specified, and unspecified)?
2. Can clinicians distinguish between ARFID and no eating pathology based on the proposed ICD-11 guidelines?
3. Some individuals present with atypical reasons for restricting eating, such as feeling uncomfortable when full. In such cases, can clinicians accurately distinguish between AN and ARFID based on the proposed ICD-11 guidelines?
4. ICD-11 has proposed that a diagnosis of AN be retained until an individual has at least 1 year of stabilized weight gain and cessation of behaviors aimed at promoting weight loss. Does this rule improve diagnostic accuracy for AN over the course of recovery?
5. Is the proposal to include subjective binge eating in ICD-11 BN clinically useful and effective in reducing residual eating disorder diagnoses?
6. Do the proposed guidelines for ICD-11 enable clinicians to accurately distinguish between BN and BED?
7. Are the proposed ICD-11 guidelines for BED clinically useful in distinguishing BED from no disorder?
8. Do the proposed ICD-11 guidelines provide sufficient clinical guidelines to distinguish BN and BED regardless of weight status?

### Participants

Participants in this study were members of the Global Clinical Practice Network (GCPN) [21]. Beginning in 2011, mental health and primary care professionals from around the globe were invited to join the Global Clinical Practice Network in order to participate in internet-based field studies of the proposed guidelines for the ICD-11 [22]. For the purpose of the present study, an internet-based protocol using the Qualtrics survey platform [23] was developed. All registered GCPN members at the time of the study were invited to participate provided (a) they were currently seeing patients or engaged in direct clinical supervision, which was operationally defined as 10 h or more per week and (b) they had identified themselves as proficient in one of the five languages of the study (Chinese, English, French, Japanese, and Spanish).

### Development of case vignettes

Vignettes were developed and validated to test the eight core study questions; that is, to test specific changes proposed for the ICD-11 as compared to the ICD-10. Members of the Feeding and Eating Disorders Working Group developed case vignettes (Table 2) based on actual clinical patient presentations that addressed the essential features being analyzed. A second, independent group of international eating disorder experts conducted confirmatory evaluations to ensure diagnostic agreement for the case narratives. These procedures follow best practices established for vignette development for such field studies [20, 24].

**Table 2:** Case vignettes with their accurate diagnoses according to either the ICD-10 or ICD-11 diagnostic guidelines

Vignette number	Key features of case vignette	Accurate diagnosis according to the ICD-10 guidelines	Accurate diagnosis according to the ICD-11 guidelines
1A	Past history of AN with amenorrhea Weight restored greater than 1 month but less than 1 year Still in treatment for AN No current weight loss behaviors but limited preoccupation with weight/shape that did not impact weight maintenance	No diagnosis/atypical anorexia nervosa	Anorexia nervosa
1B	Same as 1A, but weight restored for more than 1 year	No diagnosis	No diagnosis
1C	All key features of AN present for more than 1 month (i.e., limited food intake, and a clear fear of gaining weight or body image distortion) Individual also has amenorrhea Adolescent female	Anorexia nervosa	Anorexia nervosa
2A	Restricting food (avoidance of certain types of foods due to their sensorial characteristics, not because they were high calorie foods) and is consequently underweight Body image and fear of fatness denied and are not evident in behaviors Psychosocial functioning impaired Adolescent female	Other ED/ED unspecified/atypical AN/feeding disorder of infancy or childhood	ARFID
2B	Unusual eating habits but not diagnostic No distress Within normal weight range No psychosocial impairment	No diagnosis	No diagnosis
2C	Food restriction due to subjective somatic discomfort (does not limit specific kinds of foods, per se, just the amount) Underweight Body image and fear of fatness denied and are not evident in behaviors Adolescent female	Atypical anorexia nervosa/other ED/ED unspecified	ARFID
3A	Binge eating objectively large Compensation (purging) present Normal weight range	Bulimia nervosa	Bulimia nervosa
3B	Same symptoms and behaviors as 3A except binge eating subjectively large (perceived to be large by the individual) Slightly overweight (BMI 26)	Atypical bulimia nervosa/other ED/ED unspecified	Bulimia nervosa
3C	Similar to 3A except is obese (BMI 31)	Bulimia nervosa	Bulimia nervosa
4A	All criteria for binge eating disorder Overweight (BMI 27) Binge eating objectively large Compensation not present	Overeating associated with other psychological disturbances/atypical bulimia nervosa/other ED/ED unspecified	Binge eating disorder
4B	Overeating with no loss of control or marked distress	No diagnosis	No diagnosis
4C	Similar to 4A but obese (BMI 34)	Overeating associated with other psychological disturbances/atypical bulimia nervosa/other ED/ ED unspecified	Binge eating disorder

*AN* anorexia nervosa, *BMI* body mass index, *ED* eating disorder, *ARFID* avoidant-restrictive food intake disorder

For the purpose of evaluating the clinical utility of the ICD-11 guidelines in this study, members of the workgroup decided which ICD-10 diagnosis (or diagnoses) represented the best fit for the relevant vignettes. Because BED and ARFID are new diagnoses in ICD-11, there is not an exact comparable diagnosis in ICD-10. Thus, when applying the available options in ICD-10, a specific case could be diagnosed as “atypical” or “other specified” of “unspecified,” or, depending on the specific features of the case, as “feeding disorder of infancy or childhood” or “overeating associated with other psychological disturbances.” None of these options would fit the exact case description for conditions of BED and ARFID, but they would be the best diagnoses available using ICD-10. For these vignettes, we identified all diagnoses in the ICD-10 that could reasonably be used to diagnose these presentations and considered them “applicable.”

As for ICD-11, the generation of diagnosis for the case vignettes involved a rigorous process whereby members of the expert Working Group provided independent diagnoses for each case vignette and indicated in the case vignette each of the essential features required for that diagnosis. Any ambiguity that emerged at this stage was addressed. It was on this basis that the diagnosis considered accurate for each case vignette was defined.

### Procedures

At the time of data collection in 2014–2015, 7582 GCPN members were eligible to participate in the study and were invited. Of those, 3059 (40.3%) responded to the survey link and initiated the study. Upon entry to the study, participants were randomized to a condition in which they viewed either ICD-10 or ICD-11 clinical descriptions and diagnostic guidelines for feeding and eating disorders. They were blind to whether they were assigned ICD-10 or ICD-11 guidelines. Clinicians were then randomly assigned to one of the eight core research questions described above, which were addressed by paired-vignette comparisons. The rationale for each core diagnostic question, the description of each case vignette, and the paired vignettes used to examine each research question are described in Tables 2 and 4. Additionally, the cases were presented in counterbalanced order for each comparison. Participants used the guidelines to which they were assigned to diagnose each of the two cases presented to them. Clinical utility of the proposed ICD-11 guidelines was also compared to the ICD-10 guidelines.

After reading each of their assigned vignettes, participants selected a diagnosis from the respective diagnostic system (ICD-11 or ICD-10), with an option to enter a diagnosis other than a feeding or eating disorder (i.e., another Mental and Behavioral Disorder) if they believed that a different diagnosis was more appropriate. Participants could also indicate that no diagnosis was warranted. They were specifically asked to provide a current (as opposed to lifetime) diagnosis and could review the diagnostic guidelines and vignette while making a selection. After providing a diagnosis, participants were shown each of the essential features for their chosen diagnosis, one by one, and were asked to indicate if the clinical case described in the vignette reflected each one. After reviewing the essential features, participants had the option to change their final diagnosis. If a diagnosis was chosen that was not the diagnosis considered correct for the vignette, they were asked to articulate their reasoning (without being informed that the selected diagnosis was considered incorrect). This procedure made it possible to identify specific points of ambiguity or confusion in the classification.

Upon completion of the first vignette, each participant was presented with the second vignette and repeated the procedure described above. After selecting a diagnosis and answering the related diagnostic questions for both vignettes, participants also completed a set of questions related to the clinical utility of the diagnostic guidelines, including their ease of use, goodness of fit, and clarity.

### Statistical analysis

The study design was a 2 × 8 (diagnostic system vs. paired vignette) comparison mixed design, where the diagnostic system (ICD-10 vs. ICD-11) and the eight specific diagnostic comparisons described above were between-participant factors, with a within-participant factor comparing ratings of the two vignettes. Two-way chi-square statistics were used for bivariate comparisons and the G-square statistic [25] for three-way interactions. Data from all five languages in which the study was administered were combined in the results reported in this article.

## Results

### Participants

Of the 3059 who started the survey, 2288 (74.8%) provided complete data for inclusion in the present analysis. Participants that completed the study had approximately half a year more experience, on average (participated *M* = 13.62, *SD* = 10.20; not participated *M* = 13.08, *SD* = 10.30; *t*(7580) = 2.26, *p* < .05, *d* = 0.05). Participants represented all world regions. The largest numbers of participants came from Europe (33.0%) and the Asian portion of the Western Pacific Region (30.3%), followed by Latin America and the Caribbean (12.1% each) and the USA and Canada (10.0%). Some regions were disproportionately represented in the final sample. Participants from the Asian region of the Western Pacific (30.4% vs. 37.6%; *χ*^2^ (1) = 17.81, *p* < .001) and North American (10.0% vs. 11.8%; *χ*^2^ (1) = 4.14, *p* < .05) were underrepresented relative to the number of people invited to participate. European (33.0% vs. 28.9%; *χ*^2^ (1) = 6.69, *p* < .01), Southeast Asian (6.3% vs. 4.8%; *χ*^2^ (1) = 6.08, *p* < .05), and African (2.8% vs. 1.3%; *χ*^2^ (1) = 18.59, *p* < .001) participants were overrepresented. Male participants slightly outnumbered female participants. The majority (59.7%) were physicians (nearly all psychiatrists), and an additional 30.3% were psychologists. Most were middle-aged with approximately a decade or more of clinical experience. See Table 3 for additional details regarding demographic and other participant features.

**Table 3:** Participant demographics (*N* = 2288)

	Language group					
	All	English	Spanish	Japanese	French	Chinese
	*N (%)*	1061 (46%)	315 (14%)	340 (15%)	219 (10%)	353 (15%)
WHO global region						
Africa	64 (2.8%)	50 (4.7%)	0	0	14 (6.4%)	0
USA and Canada	229 (10.0%)	221 (20.8%)	1 (0.3%)	0	7 (3.2%)	0
Latin America/Caribbean	276 (12.1%)	43 (4.1%)	226 (71.8%)	0	7 (3.2%)	0
Eastern Mediterranean	52 (2.3%)	46 (4.3%)	0	0	6 (2.7%)	0
Europe	755 (33.0%)	484 (45.6%)	86 (27.3%)	0	185 (84.5%)	0
Southeast Asia	144 (6.3%)	144 (13.6%)	0	0	0	0
Western Pacific—Asia	695 (30.3%)	5 (0.5%)	0	337 (99.1%)	0	353 (100%)
Western Pacific—Oceania	66 (2.9%)	66 (6.2%)	0	0	0	0
Missing	8 (0.3%)	2 (0.2%)	2 (0.6%)	3 (0.9%)	1 (0.5%)	0
Male:Female	1277:985 (56:43)%	557:479 (53:47)%	153:162 (49:51)%	255:85 (75:25)%	122:96 (56:44)%	190:163 (54:46)%
Profession						
Medicine	1367 (59.7%)	515 (48.5%)	125 (39.7%)	270 (79.4%)	145 (66.2%)	312 (88.4%)
Psychology	693 (30.3%)	397 (37.4%)	161 (51.1%)	52 (15.3%)	58 (26.5%)	25 (7.1%)
Counseling	85 (3.7%)	68 (6.4%)	3 (1.0%)	3 (0.9%)	2 (0.9%)	9 (2.5%)
Nursing	49 (2.1%)	26 (2.5%)	2 (0.6%)	6 (1.8%)	11(5.0%)	4 (1.1%)
Social work	24 (1.0%)	17 (1.6%)	3 (1.0%)	1 (0.3%)	0	3 (0.8%)
Sex therapy	6 (0.3%)	6 (0.6%)	0	0	0	0
Speech therapy	2 (0.1%)	2 (0.2%)	0	0	0	0
Other	62 (2.7%)	30 (2.8%)	21 (6.7%)	8 (2.4%)	3 (1.4%)	0
*Mean (SD)*						
Age	44.52 (11.08)	46.22 (10.91)	45.96 (11.75)	44.64 (10.26)	42.62 (12.29)	39.17 (8.87)
Years of experience	13.77 (10.12)	14.60 (10.08)	16.56 (10.58)	13.31 (9.89)	13.73 (10.82)	9.29 (7.95)

### Eight core questions (Table 4)

1. Does the proposed addition of ARFID in the ICD-11 result in individuals with ARFID being more accurately distinguished from AN, and does the proposed addition of ARFID to ICD-11 reduce the number of individuals diagnosed with residual eating disorder categories (atypical, other specified, and unspecified)?

**Table 4:** Core scientific questions, rationale, vignette comparison and results

Core scientific question	Rationale	Vignette comparison ICD-11 diagnosis	Results
1. Does the proposed addition of ARFID in the ICD-11 result in individuals with ARFID being more accurately distinguished from AN, and does the proposed addition of ARFID to ICD-11 reduce the number of individuals diagnosed with residual eating disorders (atypical, other specified, and unspecified)?	The proposal to include ARFID in ICD-11 raised the research question as to whether ARFID when it is associated with underweight status can be accurately distinguished from AN using proposed ICD-11 guidelines	Vignette 1C: AN vs Vignette 2A: ARFID	ICD-11 AN DX: 96.6% accuracy ICD-10 AN DX: 93.7% accuracy *χ*^2^ (1) = 1.38, *p* = .24 ICD-11 ARFID DX: 89.9% accuracy ICD-10 ARFID DX*: 80.4% accuracy *χ*^2^ (1) = 2.34, *p* = .13 ICD-11 ARFID DX vs AN DX: *χ*^2^ (2) = 246.25, *p* < 0.001 Overall ICD-11 was equal to ICD-10, but ICD-10 “applicable” options are spread across four diagnoses* G^2^ (4) = 7.32, *p* = .16
2. Can clinicians distinguish between ARFID and no eating pathology based on the proposed ICD-11 guidelines?	The addition of a “new” diagnosis always raises the question of whether the proposed disorder can be properly distinguished from no disorder. The core research question addressed by this comparison was whether clinicians could better distinguish between ARFID and cases that should not be assigned a diagnosis based on the proposed ICD-11 guidelines as compared to the range of eating disorder residual categories in ICD-10.	Vignette 2A: ARFID vs Vignette 2B: No DX	ICD-11 ARFID DX: 88.5% accuracy ICD-10 ARFID DX*: 76.8% accuracy *χ*^2^ (1) = 6.71, *p* < .01 ICD-11 No DX: 78.4% accuracy ICD-10 No DX: 79.6% accuracy *χ*^2^ (1) = 0.17, *p* = .68. ICD-11 ARFID DX vs No DX *χ*^2^ (2) = 190.00, *p* < 0.001 Overall ICD-11 Outperformed ICD-10 *G*^2^ (4) = 17.80, *p* < 0.01.
3. Some individuals present with atypical reasons for restricting eating, such as feeling uncomfortable when full. In such cases, can clinicians accurately distinguish between AN and ARFID based on the proposed ICD-11 guidelines?	The diagnostic guidelines for a new disorder must sufficiently differentiate it from other existing disorders. We tested whether the proposed inclusion of ARFID can be clearly distinguished from AN when the rationale for restricting intake is atypical (e.g., restricting eating because of stomach fullness or bloating.	Vignette 1C: AN vs Vignette 2C: ARFID	ICD-11 AN DX: 96.7% accuracy ICD-10 AN DX: 97.0% accuracy *χ*^2^ (1) = 0.02, *p* = .89 ICD-11 ARFID DX: 87.9% accuracy ICD-10 ARFID DX**: 76.0% accuracy *χ*^2^ (1) = 6.90, *p* < 0.01 ICD-11 AN DX vs ARFID DX: *χ*^2^ (2) = 262.84, *p* < 0.001 Overall ICD-11 outperformed ICD-10 *G*^2^ (4) = 14.62, *p* < 0.01
4. ICD-11 proposes that a diagnosis of AN be retained until an individual has at least one year of stabilized sufficient weight gain and cessation of behaviors aimed at promoting weight loss. Does this rule improve diagnostic accuracy for AN over the course of recovery?	ICD-10 does not provide clear diagnostic guidance for recently weight restored individuals with AN, which results in substantial variability in whether an AN diagnosis is applied to cases that still exhibit significant symptoms but have gained weight to within a relevant weight reference (e.g., based on BMI or population quartile). ICD-11 proposes that the diagnosis of AN continue to be applied until the individual has achieved attitudinal and weight recovery for 1 year without the support of continuing care.	Vignette 1A: AN (with recovery not yet independently sustained for 1 year) vs Vignette 1B: no DX (AN with recovery independently sustained over 1 year)	ICD-11 AN DX: 84.6% accuracy for 1A ICD-11 no DX: 38.4% accuracy for 1B^Ϯ^ ICD-11 AN DX vs no DX *χ*^2^ (2) = 46.82, *p* < .001 (No independent ICD-10 comparison because this rule is new to ICD-11) Overall ICD-11 outperformed ICD-10 *G*^2^ (4)^ϮϮ^ = 31.84, *p* < 0.0001
5. Is the proposal to include subjective binge eating in ICD-11 BN clinically useful and effective in reducing residual eating disorder diagnoses?	The ICD-11 recommendation to allow subjective binge eating to fulfill a part of the diagnostic requirements for both BN and BED was based on extant data suggesting that the threshold for an objective binge episode is arbitrary and clinical reports indicating that binge size does not predict distress or impairment. Although intended to improve clinical utility, the ICD-11 inclusion of subjective binge eating could inadvertently make the diagnosis of BN or BED more difficult.	Vignette 3A: BN (with objective binge eating) vs Vignette 3B: BN (with subjective binge eating)	ICD-11 Objective BN DX: 84.3% accuracy ICD-10 Objective BN DX: 82.2% accuracy *χ*^2^ (1) = 0.23, *p* = .63 ICD-11 Subjective BN DX: 61.4% accuracy ICD-10 Subjective BN DX***: 69.6% accuracy *χ*^2^ (1) = 10.62, *p* < 0.001 ICD-11 objective BN DX vs subjective BN DX: *χ*^2^ (1) = 20.25, *p* < 0.001 Clinicians were more accurate in diagnosing BN with objective binge eating Overall ICD-11 outperformed ICD-10 *G*^2^ (2) = 10.90, *p* < 0.01.
6. Do the proposed guidelines for ICD-11 enable clinicians to accurately distinguish between BN and BED?	This question is prompted by the inclusion of the new category of BED in ICD-11.	Vignette 3A: BN vs Vignette 4A: BED	ICD-11 BN DX: 90.2% accuracy ICD-10 BN DX: 83.3% accuracy *χ*^2^ (2) = 8.73, *p* < 0.05 ICD-11 BED DX: 78.0% accuracy ICD-10 BED “equivalent” DX: 70.7% accuracy *χ*^2^ (2) = 2.05, *p* = .36 ICD-11 BN DX vs BED DX *χ*^2^ (2) = 182.50, *p* < 0.001 ICD-10 BN DX vs BED DX**** Wide variability of DX since BED does not exist in ICD-10. *χ*^2^ (2) = 152.99, *p* < 0.001 Overall ICD-11 outperformed ICD-10 *G*^2^ (4) = 11.40, *p* < 0.05
7. Are the proposed ICD-11 guidelines for BED clinically useful in distinguishing BED from no disorder?	Similar to Question 2, given the addition of BED to the ICD-11, the question arises whether the proposed disorder of BED can be properly distinguished from no disorder.	Vignette 4A: BED vs Vignette 4B: No DX	ICD-11 BED DX: 82.4% accuracy ICD-10 BED (equivalent) DX****: 72.5% accuracy *χ*^2^ (2) = 6.71, *p* < 0.05 ICD-11 No DX: 80.3% accuracy ICD-10 No Dx: 76.8% accuracy *χ*^2^ (2) = 10.54, *p* < 0.01 ICD-11 BED vs No DX: *χ*^2^ (2) = 203.40, *p* < 0.001 Overall ICD-11 outperformed ICD-10 *G*^2^ (4) = 18.24, *p* < 0.01
8. Do the proposed ICD-11 guidelines provide sufficient clinical guidelines to distinguish BN and BED regardless of weight status?	This comparison examined the impact of weight status on the diagnosis of BN and BED. According to both the proposed ICD-11 guidelines and the ICD-10 guidelines, weight status should not impact diagnosis of BN and BED. However, given that the majority of individuals who present with BED for clinical care are also overweight, this question is designed to assess whether clinicians are able to accurately distinguish between BN and BED regardless of weight status.	Vignette 3A: BN normal weight vs Vignettes 3C: BN with obesity Vignette 4A: BED slightly overweight vs Vignette 4C: BED with obesity Vignettes 3C: BN with obesity vs Vignette 4C: BED with obesity	ICD-11 BN DX with obesity: 88.5% accurate ICD-11 BN DX normal weight: 90.2% accurate *χ*^2^ (2) = 3.25, *p* = .20 ICD-11 BED DX obese: 90.5% accurate ICD-11 BED DX slightly overweight: 82.4% accurate *χ*^2^ (2) = 8.90, *p* < 0.05 ICD-11 BED DX obese vs BN DX obese *χ*^2^ (2) = 213.70, *p* < 0.001 ICD-10 BED “equivalent” DX**** with obesity: 83.2% accurate ICD-10 BED “equivalent” DX**** with slight overweight status: 70.7% accurate *χ*^2^ (2) = 7.64, *p* < 0.05 ICD-10 BN DX with obesity: 69.3% accurate ICD-10 BN DX normal weight: 83.32% accurate *χ*^2^ (2) = 8.18, *p* < 0.05 ICD-11 vs ICD-10 for BN DX with obesity *χ*^2^ (2) = 17.43, *p* < 0.001 ICD-11 vs ICD-10 for BED DC with obesity /BED “equivalent” DX with obesity *χ*^2^ (2) = 3.52, *p* = .17 Overall ICD-11 outperformed ICD-10 for obese individuals with either BN or BED *G*^2^ (4) = 21.54, *p* < 0.001

Note: *AN* anorexia nervosa, *ARFID* avoidant-restrictive food intake disorder, *BN* bulimia nervosa, *BED* binge eating disorder, *DX* diagnosis. *Accurate DX in ICD-10: atypical anorexia nervosa, feeding disorder of infancy or childhood, other eating disorder or eating disorder unspecified; **accurate DX in ICD-10: atypical anorexia nervosa, other eating disorder or eating disorder unspecified; ***accurate DX in ICD-10: atypical bulimia nervosa, other eating disorder or eating disorder unspecified; ****accurate DX in ICD-10: atypical BN, overeating associated with other psychological disturbances, other eating disorder, or eating disorder unspecified. ^Ϯ^Vignette 1B = 53.1% still diagnosed AN in ICD-11; ^ϮϮ^because of the different diagnostic labels included in ICD-11 versus ICD-10, it is not possible to have a direct, diagnosis by diagnosis comparison of the two systems, and disorders were grouped into anorexia nervosa, another diagnosis, or no diagnosis for this comparison

Clinicians were highly accurate in diagnosing AN using both the ICD-11 and the ICD-10 guidelines (the percentage of correct diagnoses for AN vignettes was 96.6% and 93.7%, respectively). The difference between systems was not significant, *χ*^2^ (1) = 1.38, *p* = .24. Clinicians assigned to the ICD-11 guidelines were able to successfully differentiate cases of ARFID from AN, *χ*^2^ (2) = 246.25, *p* < 0.001. The majority of clinicians in both the ICD-11 and ICD-10 conditions accurately diagnosed the ARFID case (89.9% and 80.4% respectively, *χ*^2^ (1) = 2.34, *p* = .13). There was no overall difference between ICD-10 and ICD-11, *G*^2^ (4) = 7.32, *p* = .16. However, because ARFID does not exist in the ICD-10, the diagnoses applied by clinicians in the ICD-10 condition were highly varied and distributed across four “applicable” options (atypical anorexia nervosa, feeding disorder of infancy or childhood, other eating disorder, or eating disorder unspecified). Thus, the addition of ARFID in ICD-11 resulted in simplifying the diagnostic landscape relative to the options available under ICD-10.

2. Can clinicians distinguish between ARFID and no eating pathology based on the proposed ICD-11 guidelines?

Using ICD-11, clinicians were able to differentiate ARFID (88.5% correct) from no diagnosis (78.4% correct), *χ*^2^ (2) = 190.00, *p* < 0.001. Using ICD-10, clinicians were also able to differentiate individuals with ARFID symptoms (although diagnoses varied because ARFID does not exist in ICD-10 as mentioned in question 1) from no diagnosis (76.8% and 79.6%, respectively), *χ*^2^ (2) = 169.50, *p* < 0.001. Clinicians using the ICD-11 were more accurate than ICD-10 for the ARFID case, *χ*^2^ (1) = 6.71, *p* < 0.01. Using both the ICD-11 and ICD-10, clinicians correctly gave no diagnosis where appropriate, *χ*^2^ (1) = 0.17, *p* = .68. When looking at overall differences across systems, clinicians using the ICD-11 outperformed those using the ICD-10, *G*^2^ (4) = 17.80, *p* < 0.01.

3. Some individuals with anorexia nervosa present with atypical reasons for restricting eating, such as feeling uncomfortable when full. Can clinicians accurately distinguish between AN and ARFID based on the proposed ICD-11 guidelines in such cases?

Clinicians using ICD-11 reliably differentiated between AN and ARFID, *χ*^2^ (2) = 262.84, *p* < 0.001. Clinicians using both ICD-10 and ICD-11 correctly diagnosed the AN case (96.7% and 97.0% respectively, *χ*^2^ (1) = 0.02, *p* = .89). However, the case that would be diagnosed with ARFID in ICD-11 resulted in multiple diagnoses of participants assigned to the ICD-10 condition. If we consider the diagnoses of atypical anorexia nervosa, other eating disorder, or eating disorder unspecified as applicable under ICD-10, clinicians still did not do as well using ICD-10 as in ICD-11 when diagnosing the same case vignette (76.0% vs. 87.9% respectively, *χ*^2^ (1) = 6.90, *p* < 0.01). Overall, the ICD-11 outperformed the ICD-10, *G*^2^ (4) = 14.62, *p* < 0.01.

4. ICD-11 proposes that a diagnosis of AN be retained until an individual has at least 1 year of stabilized weight gain and cessation of behaviors aimed at promoting weight loss. Does this rule improve diagnostic consistency for AN over the course of recovery?

The majority of clinicians (84.6%) using the ICD-11 correctly applied the new guideline for the case intended to represent AN given the fact that restoration of sufficient weight had not been sustained independent of treatment for a minimum of 1 year. Just over half (53.1%) of the clinicians using the ICD-11 incorrectly continued to apply the diagnosis of AN to the case that depicted someone who had surpassed 1 year of treatment gains and who therefore should have received no diagnosis; thus, diagnostic accuracy for the first case was higher than for the second, *χ*^2^ (2) = 46.82, *p* < 0.001. Among these individuals, there was considerable confusion about the presence or absence of specific essential features of AN in the vignette. However, all but seven recognized that the treatment gains had been maintained for at least 1 year (which according to ICD-11 would call for no diagnosis). After reviewing the diagnostic guidelines in detail, 15 of the 69 opted to change their diagnosis to “no diagnosis,” which was the correct answer. Comparing the accuracy of diagnosis utilizing ICD-11 to ICD-10, clinicians using the ICD-11 guidelines were significantly better able to distinguish between AN, another diagnosis, or no diagnosis (*G*^2^ (4) = 31.84, *p* < 0.0001), although diagnosis had to be grouped into AN, another diagnosis, or no diagnosis for this analysis.

5. Is the proposal to include subjective binge eating in ICD-11 BN clinically useful and effective in reducing residual eating disorder diagnoses?

Clinicians did not consistently apply the diagnosis of BN to the case vignette depicting an individual engaged in subjective binge eating. Participants using ICD-11 were more likely to give a diagnosis other than BN in the case of subjective binge eating when compared with the vignette describing objective binge eating (61.4% and 84.3% correct, respectively; *χ*^2^ (2) = 20.25, *p* < 0.001). Similarly, participants assigned to the ICD-10 condition were more likely to give the applicable diagnostic options when the vignette described objective binge eating (i.e., BN) compared to subjective binge eating (i.e., atypical bulimia nervosa, other eating disorder or eating disorder unspecified) 82.2% and 69.6% correct, respectively; *χ*^2^ (2) = 45.95, *p* < 0.001. Clinicians assigned to the ICD-11 condition were more accurate when diagnosing a case with subjective binge eating, *χ*^2^ (1) = 10.62, *p* < 0.001, but no different when diagnosing a case with objective binge eating, *χ*^2^ (1) = 0.23, *p* = .63. Overall, ICD-11 performed better than ICD-10, *G*^2^ (2) = 10.90, *p* < 0.01.

6. Do the proposed guidelines for ICD-11 enable clinicians to accurately distinguish between BN and BED?

The vast majority of participants correctly diagnosed the BED and BN case vignettes in ICD-11 (78.0% and 90.2%, respectively). The results indicate that participants using the ICD-11 were able to accurately distinguish between BN and BED, *χ*^2^ (2) = 182.50, *p* < 0.001. Clinicians assigned to the ICD-10 condition were highly variable in the diagnosis they chose for the case depicting binge eating without compensatory behavior: atypical BN (23.3%), overeating associated with other psychological disturbances (31.3%), other eating disorder (3.3%), eating disorder unspecified (12.7%), another diagnosis (29.3%). If the first four categories are considered as applicable options or as BED “equivalent,” as BED is not an existing category according to the ICD-10, then clinicians were able to accurately distinguish between BN and BED using both the ICD-10 and ICD-11, *χ*^2^ (2) = 2.05, *p* = .36. However, when using the ICD-10, the case depicting the syndrome of binge eating without compensatory behavior resulted in a widely variable range of diagnoses. When diagnosing BN, clinicians in the ICD-11 condition were significantly more likely to assign a correct diagnosis than those in the ICD-10 condition (90.2% vs. 83.3%, respectively), *χ*^2^ (2) = 8.73, *p* < 0.05. Clinicians using ICD-10 also differentiated the two cases, *χ*^2^ (2) = 152.99, *p* < 0.001, but overall, ICD-11 performed significantly better than ICD-10, *G*^2^ (4) = 11.40, *p* < 0.05.

7. Based on the proposed ICD-11 guidelines, can BED be reliably distinguished from non-pathological variations in eating behavior?

The majority of clinicians in the ICD-11 condition correctly diagnosed BED (82.4%) with only 1.4% failing to give this case a diagnosis, *χ*^2^ (2) = 203.40, *p* < 0.001. For the clinicians using ICD-10, 72.5% selected a binge eating disorder “equivalent” diagnosis (applicable options as mentioned in question 6) and only 7.0% failed to give this case a diagnosis. Clinicians using ICD-11 were accurate in distinguishing BED from no disorder such that most clinicians (80.3%) selected no diagnosis for the case representing no disorder. For the ICD-10 condition, 76.8% of clinicians assigned no diagnosis to the corresponding vignette, and 18.3% incorrectly assigned a BED “equivalent” diagnosis, *χ*^2^ (2) = 138.96, *p* < 0.001. Clinicians in the ICD-11 conditions were more accurate in diagnosing BED, *χ*^2^ (2) = 6.71, *p* < 0.05, and no eating disorder, *χ*^2^ (2) = 10.54, *p* < 0.01. Comparing the clinicians’ accuracy overall, ICD-11 outperformed and evidenced a cleaner pattern than ICD-10, *G*^2^ (4) = 18.24, *p* < 0.01.

8. Do the proposed ICD-11 guidelines facilitate an accurate distinction between BN and BED regardless of weight status?

Clinicians using the ICD-11 were more accurate in diagnosing BED when the case was described as clearly obese (90.5%) (BMI = 34 kg/m^2^) as compared to when the case was described as slightly overweight (BMI = 27 kg/m^2^) (82.4%), *χ*^2^ (2) = 8.90, *p* < 0.05. In the case of BN, there was no difference based on whether weight status was described as normal (90.2%) or overweight (88.5%), *χ*^2^ (2) = 3.25, *p* = .20. Overall, clinicians using ICD-11 accurately distinguished between BN and BED when the cases were described as overweight, *χ*^2^ (2) = 213.70, *p* < 0.001.

In the ICD-10 condition, clinicians showed greater accuracy in diagnosing BED “equivalent” conditions when the case was obese (83.2%) as compared to when the case was slightly overweight (70.7%, *χ*^2^ (2) = 7.64, *p* < 0.05). Also, clinicians using the ICD-10 guidelines more accurately diagnosed BN when the case was described as normal weight (83.3%) compared to when the case was described as overweight (69.3%), *χ*^2^ (2) = 8.18, *p* < 0.05. Comparing across ICD-11 and ICD-10 conditions, clinicians using the ICD-11 were more accurate than those using the ICD-10 in diagnosing BN when the case was described as obese, *χ*^2^ (2) = 17.43, *p* < 0.001. Clinicians performed equally well in diagnosing BED associated with obesity, *χ*^2^ (2) = 3.52, *p* = .17. Overall, clinicians in the ICD-11 compared to those in the ICD-10 condition provided more accurate diagnoses when a patient was described as overweight, *G*^2^ (4) = 21.54, *p* < 0.001.

### Clinical utility of the diagnoses

Clinician ratings of the clinical utility for the diagnostic guidelines of ICD-10 and ICD-11 for the conditions studied in this set of research questions are shown in Table 5. For most diagnoses, the pattern of results for ICD-11 as compared to ICD-10 was the same. ICD-11 was rated more favorably than ICD-10 for each diagnosis in terms of (1) how easy the diagnostic categories were to use, (2) how well the guidelines fit the case vignettes, and (3) how clear the guidelines were.

**Table 5:** Clinical utility ratings for ICD-11 categories as compared to closest ICD-10 categories

Diagnostic category	Not at all	Somewhat	Quite	Extremely	*Quite + Extremely	
Ease of use *N (%)*						
ICD-11 AN	2 (0.5%)	62 (14.7%)	223 (52.8%)	135 (32.0%)	358 (84.8%)	*χ*^2^ (3) = 10.17, *p* < 0.05
ICD-10 AN	12 (3.5%)	56 (16.2%)	170 (49.1%)	108 (31.2%)	278 (80.3%)	
ICD-11 BN	6 (1.5%)	50 (12.3%)	188 (46.4%)	161 (39.8%)	349 (86.2%)	*χ*^2^ (3) = 47.25, *p* < 0.001
ICD-10 BN	12 (3.5%)	82 (24.2%)	182 (53.7%)	63 (18.6%)	245 (72.3%)	
ICD-11 BED	2 (0.6%)	32 (9.6%)	184 (55.1%)	116 (34.7%)	300 (89.8%)	*χ*^2^ (3) = 68.24, *p* < 0.001
ICD-10 Overeating	13 (7.6%)	47 (27.5%)	94 (55.0%)	17 (9.9%)	111 (64.9%)	
ICD-11 ARFID	8 (2.0%)	51 (13.0%)	219 (55.7%)	115 (29.3%)	334 (85.0%)	*χ*^2^ (3) = 21.63, *p* < 0.001
ICD-10 Atypical AN	5 (4.3%)	53 (28.6%)	83 (44.9%)	44 (23.8%)	127 (68.7%)	
Goodness of fit *N (%)*						
ICD-11 AN	0 (0%)	53 (12.6%)	238 (56.4%)	131 (31.0%)	369 (87.4%)	*χ*^2^ (3) = 14.07, *p* < 0.01
ICD-10 AN	6 (1.7%)	66 (19.1%)	177 (51.2%)	97 (28.0%)	274 (79.2%)	
ICD-11 BN	6 (1.5%)	44 (10.9%)	197 (48.6%)	158 (39.0%)	355 (87.6%)	*χ*^2^ (3) = 69.35, *p* < 0.001
ICD-10 BN	1 (0.3%)	95 (28.0%)	190 (56.0%)	53 (15.6%)	243 (71.6%)	
ICD-11 BED	2 (0.6%)	97 (29.0%)	175 (52.4%)	118 (35.3%)	293 (87.7%)	*χ*^2^ (3) = 33.28, *p* < 0.001
ICD-10 Overeating	9 (5.3%)	52 (30.4%)	90 (52.6%)	20 (11.7%)	110 (64.3%)	
ICD-11 ARFID	3 (0.8%)	44 (11.2%)	241 (61.3%)	105 (26.7%)	346 (88.0%)	*χ*^2^ (3) = 22.13, *p* < 0.001
ICD-10 Atypical AN	2 (1.1%)	49 (26.5%)	94 (50.8%)	40 (21.6%)	134 (72.4%)	
Clarity and understandability *N (%)*						
ICD-11 AN	2 (0.4%)	46 (10.3%)	229 (51.2%)	170 (38.0%)	399 (89.2%)	*χ*^2^ (3) = 27.71, *p* < 0.001
ICD-10 AN	11 (2.8%)	80 (20.2%)	194 (49.0%)	111 (28.0%)	305 (77.0%)	
ICD-11 BN	4 (1.0%)	49 (11.7%)	215 (51.4%)	150 (35.9%)	365 (87.3%)	*χ*^2^ (3) = 47.05, *p* < 0.001
ICD-10 BN	11 (2.9%)	92 (24.5%)	206 (54.9%)	66 (17.6%)	272 (72.5%)	
ICD-11 BED	1 (0.3%)	47 (11.7%)	213 (53.8%)	135 (34.1%)	348 (87.9%)	*χ*^2^ (3) = 28.72, *p* < 0.001
ICD-10 Overeating	8 (4.2%)	53 (28.0%)	90 (47.6%)	38 (20.1%)	128 (67.7%)	
ICD-11 ARFID	8 (1.8%)	42 (9.6%)	232 (52.8%)	157 (35.8%)	389 (88.6%)	*χ*^2^ (3) = 22.18, *p* < 0.001
ICD-10 Atypical AN	3 (1.4%)	53 (25.6%)	95 (45.9%)	56 (27.1%)	151 (73.0%)	

*AN* anorexia nervosa, *BN* bulimia nervosa, *BED* binge eating disorder, *Overeating* overeating associated with other psychological disturbances, *ARFID* avoidant-restrictive food intake disorder. *Quite + Extremely column provided for comparison only; not included in the statistical analysis

## Discussion

This vignette-based, case-controlled study found that the recommended changes to the ICD-11 diagnostic guidelines for eating disorders generally improved diagnostic accuracy and clinical utility as compared to the existing ICD-10 guidelines. The experimental design of this study facilitated rigorous comparisons of the guidelines when applied by mental health professionals around the world. The addition of the new categories of BED and ARFID significantly improved diagnostic consistency relative to ICD-10. Further, for all diagnostic categories, clinicians rated the ICD-11 guidelines significantly more favorably than ICD-10 in terms of their clinical utility, including ease of use, goodness of fit, diagnostic confidence, and clarity of the guidelines.

The study highlighted several ways in which the initially proposed guidelines needed to be improved and provided direction that guided further refinement of the ICD-11 guidelines [21]. This investigation was also useful in highlighting key issues that will need to be integrated into training efforts as the ICD-11 is adopted around the world.

First, clinicians had some difficulty determining when to consider a person with a diagnosis of AN to be recovered and discontinue use of the diagnosis of AN relative to weight status. This is a longstanding clinical conundrum, given that weight status plays such a central role in the clinical presentation of AN, and individuals with AN can gain weight despite on-going and significant attitudinal and behavioral disturbances. The proposal to extend the diagnosis of AN until an individual has sustained recovery, i.e., achieved healthy weight and cessation of behaviors aimed at reducing body weight without the support of on-going treatment, is conceptually consistent with clinical practice but, as indicated by our results, difficult to operationalize. Alternatively, it may have been that clinicians did not apply the proposed guidelines accurately because they did not realize that they were being asked to assign the “current” diagnosis for the case vignette. It is notable that in follow-up inquiries, among those clinicians who initially applied the diagnostic guidelines inaccurately, virtually all of them changed their diagnosis after the item-by-item analysis. This suggests that training on this guideline will be of significant benefit and that clinicians can accurately apply the guideline when it is brought to their attention.

The definition of recovery in AN was refined in the final guidelines by adding additional qualifiers related to underweight status. Specifically, the qualifier “Anorexia Nervosa in recovery with normal body weight” was added to the qualifiers for underweight status. This qualifier is applied as follows: “Among individuals who are recovering from Anorexia Nervosa who have reached a healthy body weight, the diagnosis should be retained until a full and lasting recovery is achieved. This includes maintenance of a healthy weight and the cessation of behaviors aimed at reducing body weight independent of the provision of treatment (e.g., for at least 1 year after intensive treatment is withdrawn).”

The second finding that resulted in revision to the guidelines pertains to subjective binge eating. Results from the current study indicate that further guidance is necessarily related to the inclusion of subjective binge eating in conferring a diagnosis of BN. Again, in clinical practice, descriptions of the size of binge eating episodes vary [26] and individuals with patterns of subjective binge eating and purging describe significant distress and indicators of psychopathology and severity are the same from individuals who describe objective binge eating [27–30]. Thus, there is a strong clinical case for applying the diagnosis of BN for these individuals. Given the results of this study, the guidelines for the assessment of binge eating in BN and BED were further elaborated in the “Additional Features” section of the guidelines to make it clear that subjective experiences of loss of control over eating and related distress are pathognomonic features of binge eating, even when not consuming an objectively large amount of food.

Specifically, in the “Additional Features” sections for both BN and BED, it is stated: "Binge eating episodes may be “objective,” in which the individual eats an amount of food that is larger than what most people would eat under similar circumstances, or “subjective,” which may involve eating amounts of food that might be objectively considered to be within normal limits but are considered large by the individual. In either case, the core feature of a binge eating episode is the experience of loss of control over eating". Again, we believe that training clinicians on this guideline will be of utmost importance since it explicitly differs from the definition of binge eating in ICD-10 and DSM-5.

Third, the findings from this study are consistent with clinical reports that clinicians tend to associate BED with obesity, probably in part because the majority of individuals who present for treatment for BED are overweight or obese [31]. The clinical description of BED in ICD-11 explicitly states that weight is not a determinative clinical feature of this disorder. To underscore the distinction between BED and weight status, in the section “Boundaries with Other Disorders and Conditions (Differential Diagnosis),” a specific section has been added as follows: “Boundary with obesity: Obesity is a common consequence of Binge Eating Disorder, and should be recorded separately. However, obese individuals who report overeating patterns that do not meet the definition of binge eating should not be diagnosed with Binge Eating Disorder”. Given the practical reality, training materials for feeding and eating disorders will need to also underscore this point.

The inclusion of the additional diagnoses of BED and ARFID and the broadening of diagnostic requirements of BN to include some formerly subthreshold cases have important clinical and public health implications given that currently the majority of eating disorder diagnoses fall in the residual “other specified” or “unspecified” categories in clinical practice. With the inclusion of BED and ARFID, the results of this study suggest that there will be fewer “other specified” or “unspecified” eating disorders. We also anticipate that many individuals who are suffering from an eating disorder will more readily be able to secure treatment. Research shows that subthreshold cases often have similar levels of impairment and can develop more severe behavioral presentations over time [32–34]. The changes in the proposed ICD-11 diagnostic guidelines may help to facilitate more specific diagnoses that will guide appropriate treatment. With earlier diagnosis and treatment, we expect to prevent progression to greater severity of illness presentation and to reduce corresponding loss of function or years of life.

Finally, the present study supported the clinical utility for both schemes. However, ICD-11 was regarded by clinicians as an easier scheme to use and as having an overall clearer description of disorders and a better fit for the clinical vignettes in this study, with results indicating favorable responses of “quite” or “extreme” for these aspects of the clinical utility reaching above 85% of ratings for diagnoses in ICD-11 (Table 5). Overall, findings from this study are in line with other research that examined the clinical utility of the ICD-11 guidelines for high burden mental health disorders [35].

This is the first time in the revision of either the ICD or the DSM that a rigorous research program was pursued to systematically evaluate the impact and clinical utility of proposed changes in guidelines. The use of technology through the engagement of the Global Clinical Practice Network and the utilization of a rigorous experimental case-vignette case-control design enabled us to gather empirical data on the proposed guidelines for feeding and eating disorders, and make further changes in the recommendations prior to the finalization and publication of the ICD-11 guidelines. Because we conducted an item-by-item analysis whenever a clinician made a diagnosis that was not accurate according to the expert standard, we were able to utilize the additional feedback from participants to guide further refinement of the guidelines.

This study engaged clinicians from around the world. Every WHO region was represented, and the study was conducted in five languages [21]. The case-controlled vignette-based study methodology enabled us to evaluate the guidelines by controlling for variability associated with clinical presentations. The vignettes were developed based on actual clinical cases and most participants reported that the case vignettes were similar to the individuals they see in clinical practice. Additionally, members of the Work Group who consulted on the creation of the case vignettes represent a variety of countries, ensuring that a range of cultural perspectives was included in vignette development.

The development of the ICD-11 is notable in that this is the first time that empirical findings regarding clinical utility and global applicability of a diagnostic classification will inform further revision of the diagnostic guidelines prior to their formal adoption. These methods increase ICD-11’s ability to provide guidelines that are truly relevant and broadly applicable in real clinical practice around the globe. The findings from this study indicate that the ICD-11 will provide significantly improved guidelines for the disorders within the Feeding and Eating Disorders Category.

### Limitations

The present study used standardized case descriptions in the form of vignettes and did not involve the application of the guidelines to a real clinical sample. Therefore, the result of this study should be generalized to individual patients with caution. Nonetheless, vignettes were developed and validated by clinical experts drawing upon real cases which expert raters considered to be valid and therefore can be treated as a useful simulation of clinical decision-making within these limitations [20].

Regarding generalizability, this was a truly global, multilingual, multidisciplinary study, with vignettes and guidelines designed to be cross-culturally applicable. Nonetheless, care should always be taken when generalizing results to specific (local) populations that may differ from the general (global) sample.

## Conclusion

Overall, the results in this study indicate that the proposed ICD-11 diagnostic guidelines for eating disorders represent a significant improvement over ICD-10. Clinicians report that the ICD-11 has high clinical utility; the additional diagnostic categories appear to be widely understood and are expected to increase the clinical accuracy in the diagnosis of feeding and eating disorders. These improvements in diagnosis have the potential to facilitate the organization and delivery of services and to achieve better clinical outcomes over time.

## Abbreviations

AN: Anorexia nervosa
APA: American Psychiatry Association
ARFID: Avoidant-restrictive food intake disorder
BED: Binge eating disorder
BN: Bulimia nervosa
DSM-5: Diagnostic and Statistical Manual of Mental Disorders 5th Edition
EDs: Eating disorders
FEDs: Feeding and eating disorders
GCPN: Global Clinical Practice Network
ICD: International Classification of Diseases and Related Health Problems
WHO: World Health Organization
